# Inactivation of an Indonesian isolate of foot-and-mouth disease virus using formaldehyde

**DOI:** 10.14202/vetworld.2024.1190-1195

**Published:** 2024-06-02

**Authors:** Talenta Miracle Tobing, Fedik Abdul Rantam, Thomas Valentinus Widiyatno, Martia Rani Tacharina, Jola Rahmahani, Nusdianto Triakoso, Suryo Kuncorojakti, Heni Puspitasari, Helen Susilowati, Diyantoro Diyantoro, Fadia Azzahra, Yudha Kurniawan, Ahmad Aswin, Edy Budi Susila

**Affiliations:** 1 Undergraduate Student of Veterinary Medicine, Faculty of Veterinary Medicine, Universitas Airlangga, Surabaya, East Java, Indonesia; 2 Division of Veterinary Microbiology, Faculty of Veterinary Medicine, Universitas Airlangga, Surabaya, East Java, Indonesia; 3 Research Center for Vaccine Technology and Development, Institute of Tropical Disease, Universitas Airlangga, Surabaya, East Java, Indonesia; 4 Division of Veterinary Pathology, Faculty of Veterinary Medicine, Universitas Airlangga, Surabaya, East Java, Indonesia; 5 Division of Veterinary Clinical Science, Faculty of Veterinary Medicine, Universitas Airlangga, Surabaya, East Java, Indonesia; 6 Division of Veterinary Anatomy, Faculty of Veterinary Medicine, Universitas Airlangga, Surabaya, East Java, Indonesia; 7 Institute of Tropical Disease, Universitas Airlangga, Surabaya, East Java, Indonesia; 8 Department of Health, Faculty of Vocational Studies, Universitas Airlangga, Surabaya, East Java, Indonesia; 9 Magister Program in Vaccinology and Immunotherapeutic, Faculty of Veterinary Medicine, Universitas Airlangga, Surabaya, East Java, Indonesia; 10 Pusvetma Veterinary Farma Big Center, Directorate General of Livestock and Animal Health, Ministry of Agriculture, Indonesia

**Keywords:** foot-and-mouth disease virus, formaldehyde inactivation, vaccine development

## Abstract

**Background and Aim:**

Foot-and-mouth disease (FMD) is a highly contagious viral disease that endangers livestock and the environment with significant economic consequences. This study aimed to validate the inactivation of the Indonesian isolate of foot-and-mouth disease virus (FMDV) with various formaldehyde concentration.

**Materials and Methods:**

The experiment started with FMDV being adapted on BHK-21 cells until cytopathic effects (CPE) appeared. The biological titer of the virus was determined using the 50% tissue culture infectious dose (TCID50) assay. The virus was inactivated by exposing the isolate to different formaldehyde (FA) concentrations (0.025%, 0.05%, 0.1%, and 0.2%) at 37°C for 24 h, and residual infectivity was assessed using CPE scoring of reinoculated BHK-21 cells.

**Results:**

72 h post-inoculation, the virulence of the FMDV isolate was indicated by complete CPE on BHK-21 monolayer cells, with a TCID50 value of 109/mL; CPE scoring did not signify significant differences (p < 0.05) among 0.025%, 0.05%, 0.1%, 0.2% FA, and the negative control. All treatment groups showed significant differences (p < 0.05) from the positive control (C+). FA concentrations inactivated the FMDV isolate under the given conditions. 0.025% and 0.05% FA continued to display CPE through the third passage, while 0.2% FA did not significantly differ from 0.1% FA (p > 0.05). 0.1% FA is the optimal concentration for safely and effectively inactivating FMDV.

**Conclusion:**

All of the formaldehyde concentrations can completely inactivate the FMDV isolate, with the most optimal and safe concentration being 0.1%.

## Introduction

Foot-and-mouth disease, a highly contagious transboundary disease, has substantial socioeconomic and public health implications [1]. Foot-and-mouth disease virus (FMDV), an RNA virus with a single-stranded, positive genome, exhibits swift genetic evolution, multiple transmission avenues, a wide host range of cloven-hoofed animals, and expensive preventive methods, and has become a global issue [2]. Foot-and-mouth disease (FMD) control strategies heavily rely on vaccination. In the past and present, inactivated vaccines have been preferred for FMD outbreaks worldwide due to their safety, ability to induce protective immune responses, long-lasting immunity, rapid response, and cost-effective mass production [3].

Formaldehyde effectively inactivates both RNA and DNA viruses by forming covalent bonds between proteins through cross-linking, retaining antigenic structure, and immunogenicity. FA, which is licensed for several viral inactivated vaccines, offers advantages such as widespread availability, cost-effectiveness, and ease of use [4]. Stated by Rodríguez and Grubman [5], immunization with FA-inactivated FMD vaccines provides robust protection against FMDV infection for up to 136 days (19/20 weeks) post-boost [5]. 0.025% FA-inactivated South American FMDV remains stable at 37°C for 40 h [6], while 0.12% FA effectively inactivates FMDV serotype O (Pakistan strain) and induces detectable antibodies at 37°C for 24 h [7]. In 2022, FMD cases of the sub-lineage O/ME-SA/Ind-2001e were emerged in East Java, Indonesia. This sub-lineage, recognized for its high immunogenicity and excellent survival rates [8], is a promising Asian FMD vaccine candidate. The use of authorized multivalent inactivated vaccines matching the serotype O in response to the outbreak was endorsed by the Director General of Livestock and Animal Health, Ministry of Agriculture of the Republic of Indonesia. The vaccines did not originate from Indonesian FMDV isolates that had been inactivated [9]. The need to choose the appropriate vaccine strain is underlined because of the seven unique FMDV serotypes (O, A, C, SAT1, SAT2, SAT3, and Asia-1) resulting from the virus’s rapid mutation rate [10].

Imported vaccines are not only expensive, but they are also derived from viral strains distinct from those present in Indonesia. The inefficiency of FMD vaccines arises from heightened costs due to insignificant antigen-vaccine match and temperature-sensitivity above 8°C, which mainly impacts tropical regions [11].

This study aims to create a foundation for an inactivated FMD vaccine using the provided isolate due to the restrictions of existing vaccines for this particular strain.

## Materials and Methods

### Ethical approval

All research methods and practices and the use of experimental animals have been approved by the Animal Care and Use Committee, Faculty of Veterinary Medicine, Airlangga University, Surabaya, Indonesia, with ethical approval number: 2.KEH.101.06.2023.

### Study period and location

This study was conducted from January to December 2023. The entire research process took place in Biosafety Level-2 (BSL-2) Laboratory of Research Center for Vaccine Technology and Development of Institute of Tropical Disease, Universitas Airlangga in Surabaya, Indonesia.

### Experimental design

The cryopreserved Serotype O FMDV in this research was isolated from naturally FMD-infected cattle in Lamongan, East Java, Indonesia and cultured on a 100^th^ passage baby hamster kidney-21 (BHK-21) monolayer cell after being stored in an ultra-low temperature freezer.

The media was prepared with minimum essential media (MEM) culture media (Gibco™, New York, USA), fetal bovine serum (FBS) (Gibco), amphotericin B (1%), and pen strep (1%). The IR AutoFlow NU-8500 CO_2_ water-jacketed incubator by NuAire (Plymouth, MN, USA) was maintained at 37°C and 5% CO_2_ during the experimentation. Media and reagents were allowed to reach room temperature before use. The isolate was exposed to four different concentrations of FA (0.025%, 0.050%, 0.1%, and 0.2%) for inactivation within a 24 h period at 37°C with continuous agitation. The inactivated virus was reinoculated onto a confluent BHK-21 monolayer and incubated for 3 days, followed by three passages. Under the Nikon Eclipse Ts2 FL Inverted Diascopic Epi-Florescence Microscope + DS-Qi2 Cam (Tokyo, Japan), the CPE of inactivated viruses was observed and scored daily per well.

### Virus propagation

Once confluent and contamination-free, the old media is discarded. The virus was thawed and added to the flask. The monolayer was incubated with the flask tilted for 1 h to ensure even spread [12]. Once CPE was achieved, growth media with 5% FBS was added and the flask was incubated further. The viral supernatant was stored in an ultra-low temperature freezer for future utilization [13].

### 50% tissue culture infectious dose (TCID50) assay

A trypsinized cell suspension with trypan blue solution (0.4%) was placed onto a hemocytometer square on an inverted microscope [14]. The viability of cells was determined by counting them under a 40× microscope lens.


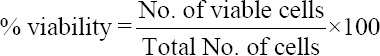


The volume of viable cells was counted and 0.5 × 10^4^ cells were seeded per well of a NEST® 96-well polystyrene non-pyrogenic sterile cell culture plate (Nest Scientific, USA) [15]. The virus inoculum was diluted tenfold serially from 10^-1^ to 10^-10^ [11]. Following cell confluence, eight replications, including controls, were carried out for each viral dilution. After 1 h of incubation to allow contact between the virus and monolayer, 5% FBS supplemented overlay media was added to each well. The media was removed, washed with Dulbecco’s phosphate-buffered saline (DPBS, Verviers, Belgium) 1×, stained with crystal violet solution, and air dried after observing CPE [7]. Wells that exhibit CPE were recorded and TCID50 was calculated [16]:


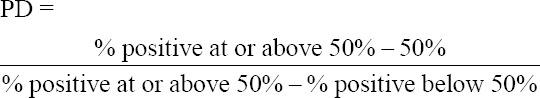


Calculation of proportionate distance (PD) between dilution above and below 50% endpoint:

Calculation of 50% endpoint titer (TCID50/0.1 mL):

10 log total dilution above 50% - (PD*log [dilution factor])

### Inactivation of FMDV using FA

A beaker glass with no FBS growth media was kept atop a Thermo Scientific™ Cimarec+™Stirring Hotplate (Thermo Scientific, USA) set at 37°C and agitated gently. Virus inoculum was added, followed by FA (0.025%, 0.050%, 0.1%, and 0.2%) in gradual increments. The beaker glass was shaken at 37°C in the NuAire AutoFlow NU-8500 CO_2_ incubator with a 5% CO_2_ level overnight [7, 17].

### Validation of FMDV inactivation

24-well NEST® plates (NEST Scientific) were prepared in advance using confluent BHK-21 cells. The old medium was replaced with fresh medium before inoculating FMDV with six repetitions per FA concentration. A culture plate with negative and positive controls is kept distinct. The plates were then incubated for 1 h and the media were discarded before adding growth media containing 5% FBS to each well [7]. The contents of the wells were passaged every 3 days until the third passage. Every day, the cells were checked for cytopathic and other effects. CPE severity levels were assessed on a scale from zero to four, where zero represented no observable CPE and four denoted 75%–100% observable CPE [18]. The third passage effectively inactivated the virus, as no CPE was detected at its end [19].

### Statistical analysis

The Kruskal–Wallis and Mann–Whitney tests were utilized in Statistical Package for the Social Sciences version 20 (IBM Corp., NY, USA) for analyzing the CPE scorings. The CPE scores were statistically analyzed using Microsoft® Excel® (Microsoft Office, Washington, USA). The biological titer of the FMD serotype O isolate was calculated to be 10^9^/mL using Reed and Muench formula [16].

## Results

### Viral propagation

**Figure-1:**
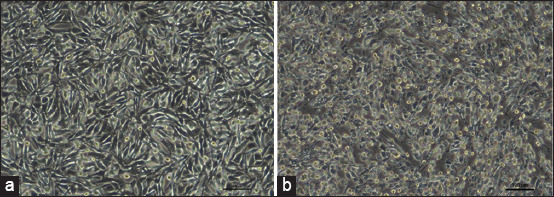
Baby hamster kidney-21 cells under 100× magnification. (a) Normal cell before virus inoculation, (b) 72 h post-viral inoculation.

Starting from the first passage, confluent BHK-21 monolayer cells exhibited CPE upon inoculation with the nontoxic FMDV isolate. The isolation of FMDV from BHK-21 cells was confirmed by the emergence of CPE, which is characterized by cell rounding, flattening, disruption of intercellular junctions, and eventual cell death. At 24 h post-infection, distinctive CPE emerged, causing cell monolayer detachment within 72 h (Figure-1).

### Biological titer determination

The TCID50 assay was performed by making tenfold serial dilutions of the viral inoculum, ranging from 10^1^ to 10^10^ with eight replications in a NEST® 96-well polystyrene non-pyrogenic sterile cell culture plate. The infectious FMDV titer was determined according to Table-1.

**Table-1:** Result of TCID50 assay.

Dilution of foot-and-mouth disease virus	No. of wells with CPEs	No. of wells without CPEs	Positive CPE ratio	% of infected (ratio × 100%)
10^1^	8	0	8/8	100
10^2^	8	0	8/8	100
10^3^	8	0	8/8	100
10^4^	8	0	8/8	100
10^5^	8	0	8/8	100
10^6^	8	0	8/8	100
10^7^	8	0	8/8	100
10^8^	5	3	5/8	62.5
10^9^	4	4	4/8	50
10^10^	1	7	1/8	12.5

CPE=Centrifuge pellet efficacy, TCID=50% Tissue culture infectious dose

### Validation of viral inactivation

**Figure-2:**
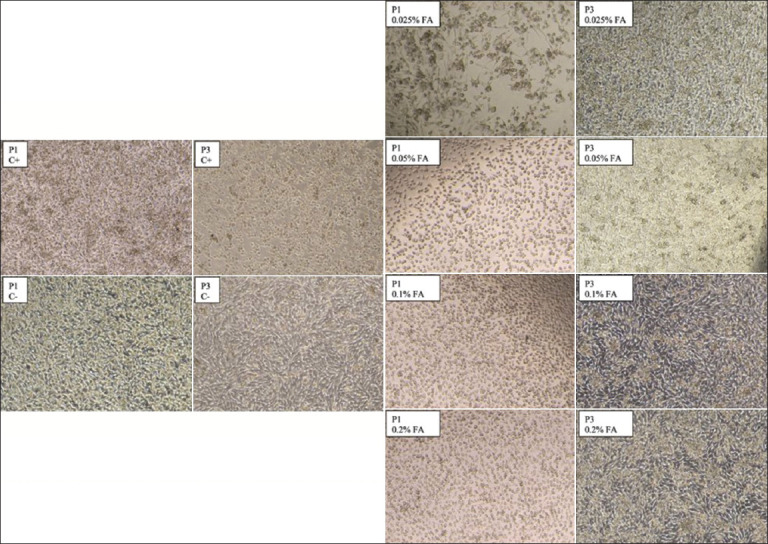
Viral inactivation validation results between treatment groups.

The residual infectivity of inactivated FMDV was assessed by passing it through three confluent layers of BHK-21 cells [19]. Due to overseeding, the cells overlapped on all wells, obscuring individual cell boundaries and complicating the analysis of single-cell morphology. FA-inactivated FMDV initially induced a transient cytopathic effect in cells. Upon further passaging, the cell’s morphology and growth patterns returned to normal. 0.1% and 0.2% FA treatments showed a quicker reversal effect compared to 0.025% and 0.05% FA, and the positive control displayed CPE, verifying the presence of suitable conditions for viral infection and replication. The negative control’s morphological normalcy confirms the system’s functionality (Figure-2).

The score ranged from zero, signifying no CPE, to four representing the most severe CPE for the inactivated virus’s CPE [18]. The most substantial alteration in CPE scoring is demonstrated in Figure-2, primarily occurring on the 1^st^ day of the second passage. 0.025% and 0.05% FA-inactivated viruses displayed CPE on day 1 of the third passage; CPE was undetectable for 0.1% and 0.2% FA-inactivated viruses. Significant differences in CPE (p < 0.05) were observed between the positive control and other treatments; no significant differences (p > 0.05) were detected between the negative control and all treatment groups. 0.025% and 0.05%, as well as 0.1% and 0.2% FA, showed no significant differences in CPE scoring (p < 0.05) between treatment groups.

## Discussion

### Biological titer of the isolate

The FMDV enters cells through macropinocytosis, leading to rapid lysis with no plaque formation [20, 21]. To identify the dilution causing CPE in 50% of the wells, the TCID50 assay was performed using infected cell cultures at a set time point. The assay evaluated the extent of CPE from viral infection. The Indonesian isolate of serotype O FMDV had a TCID50 value of 10^9^/mL. Previous studies [7, 22] found TCID50 values lower for FMDV Pakistan serotype O (106.74/mL and 108.5/mL, respectively).

### Viral inactivation and inactivation validation

The FA concentration was initially set at the lowest identified level of 0.025% according to McKercher *et al.*, [6] and was subsequently raised in increments by a factor of two up to 0.2%. After the initial triggering of CPE by FA-inactivated FMDV, cells regained normal morphology and growth in subsequent passages (Figure-2). Cell passaging supplies fresh nutrients and a new environment, reducing prior culture stress or toxicity [23]. The observed cell shrinkage morphological changes in the treatment groups could be credited to FA’s fixative properties [24]. In the following passages, the experimental cells regained their normal morphology and growth patterns. The absence of viral capsid (CPE) by the end of the third FA-inactivated FMDV passage implies complete inactivation with all used FA concentrations [19]. In later passages, the positive control exhibited faster CPE development compared to its initial, slow growth. While the negative control retained normal cell shape, the experimental group exhibited altered morphology. The control groups verified the experiment’s effectiveness.

**Figure-3:**
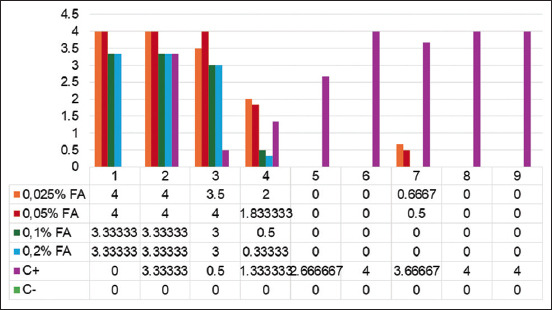
CPE scoring of viral inactivation. The y-axis represents the average CPE scoring for each treatment per day CPE. The x-axis represents the passage number (P) and day (D) after inactivated virus reinoculation. CPE=Centrifuge pellet efficacy.

Inactivated virus CPE scoring revealed a significant difference (p < 0.05) between the positive control and other treatments, but no significant difference (p > 0.05) was observed between the negative control and all treatment groups. 0.025%, 0.05%, 0.1%, and 0.2% FA showed no significant difference (p < 0.05) in CPE scoring. Although no CPE was observed in the FMDV stocks after the third passage, a warning is necessary for the 0.025% and 0.05% FA concentrations due to residual CPE present before the third passage (Figure-3). Moreover, 0.2% FA exhibited no significant difference (p > 0.05) compared to 0.1% FA throughout the passaging, potentially excessively disrupting the immunogenic viral protein structure, considering the highest known FA concentration used in previous FMDV inactivation studies is 0.12% [7]. 0.1% FA ensures optimal and safe inactivation of FMDV. To generate immunity against a virus, vaccine development necessitates the inclusion of immunogenic viral proteins. Further analysis using techniques such as SDS-PAGE and western blotting [25] is necessary to evaluate the structure of FMDV proteins post-inactivation.

## Conclusion

At 37°C, FMDV serotype O isolate is fully inactivated by FA concentrations of 0.025%, 0.05%, 0.1%, and 0.2% within 24 h. 0.1% FA is the most optimal and safe concentration for inactivating FMDV. Assessing the viral protein structure of inactivated FMDV through further research is crucial for safe veterinary vaccine production.

## Authors’ Contributions

FAR and JR: Conceived and designed the study. HP, TMT, FA, YK, AA, and EBS: Collected samples. TMT, FAR, SK, HS, DD, FA, YK, and AA: Performed the experimental works. FAR, TMT, TVW, MRT, and NT: Analysed and interpreted the data. TMT, FAR, and DD: Drafted and revised the manuscripts. All authors have read, reviewed, and approved the final manuscript.
